# Global Transcriptome and Deletome Profiles of Yeast Exposed to Transition Metals

**DOI:** 10.1371/journal.pgen.1000053

**Published:** 2008-04-25

**Authors:** Yong Hwan Jin, Paul E. Dunlap, Sandra J. McBride, Hanan Al-Refai, Pierre R. Bushel, Jonathan H. Freedman

**Affiliations:** 1 Nicholas School of the Environment and Earth Sciences, Duke University, Durham, North Carolina, United States of America; 2 Laboratory of Molecular Toxicology, National Institute of Environmental Health Sciences, NIH, Research Triangle Park, North Carolina, United States of America; 3 Biostatistics Branch, National Institute of Environmental Health Sciences, NIH, Research Triangle Park, North Carolina, United States of America; Washington University in St. Louis School of Medicine, United States of America

## Abstract

A variety of pathologies are associated with exposure to supraphysiological concentrations of essential metals and to non-essential metals and metalloids. The molecular mechanisms linking metal exposure to human pathologies have not been clearly defined. To address these gaps in our understanding of the molecular biology of transition metals, the genomic effects of exposure to Group IB (copper, silver), IIB (zinc, cadmium, mercury), VIA (chromium), and VB (arsenic) elements on the yeast *Saccharomyces cerevisiae* were examined. Two comprehensive sets of metal-responsive genomic profiles were generated following exposure to equi-toxic concentrations of metal: one that provides information on the transcriptional changes associated with metal exposure (transcriptome), and a second that provides information on the relationship between the expression of ∼4,700 non-essential genes and sensitivity to metal exposure (deletome). Approximately 22% of the genome was affected by exposure to at least one metal. Principal component and cluster analyses suggest that the chemical properties of the metal are major determinants in defining the expression profile. Furthermore, cells may have developed common or convergent regulatory mechanisms to accommodate metal exposure. The transcriptome and deletome had 22 genes in common, however, comparison between Gene Ontology biological processes for the two gene sets revealed that metal stress adaptation and detoxification categories were commonly enriched. Analysis of the transcriptome and deletome identified several evolutionarily conserved, signal transduction pathways that may be involved in regulating the responses to metal exposure. In this study, we identified genes and cognate signaling pathways that respond to exposure to essential and non-essential metals. In addition, genes that are essential for survival in the presence of these metals were identified. This information will contribute to our understanding of the molecular mechanism by which organisms respond to metal stress, and could lead to an understanding of the connection between environmental stress and signal transduction pathways.

## Author Summary

Environmental and human health threats are posed by contamination from transition metals. A variety of pathologies are associated with exposure to supraphysiological concentrations of essential metals and to non-essential metals and metalloids. To defend against metal toxicity, sophisticated defense mechanisms have evolved. Although many of the genes and regulatory pathways have been identified, the consequence of metal exposure on a systematic level has not been examined. To better define the mechanism involved in the metal response, we examined the effects of zinc, cadmium, mercury, copper, silver, chromium, and arsenic on gene expression in the yeast *Saccharomyces cerevisiae*. In addition, the roles of ∼4,500 non-essential genes in protecting yeast from metal toxicity were determined. Data analyses suggest that the chemical properties of the metal are major determinants in defining its biological effect on cells. Furthermore, cells may have developed common or convergent regulatory mechanisms to accommodate metal exposure. Several evolutionarily conserved regulatory pathways were identified that link metal exposure, disruption of normal metabolism and gene expression. These results provide a global understanding of the biological responses to metal exposure and the stress response.

## Introduction

Throughout the world, environmental and human health threats are posed by contamination from transition metals. Metals can be introduced into the environment through both natural and anthropogenic routes [1]. Human exposure routes include absorption through the skin, ingestion, and inhalation [2]. A variety of pathologies are associated with exposure to supraphysiological concentrations of essential metals (copper, chromium, zinc) and to non-essential metals and metalloids (cadmium, mercury, silver, arsenic) including cancer, organ damage, central nervous system disorders, cognitive dysfunction and psychological disorders, and birth defects [3].

The molecular mechanisms linking metal exposure to human pathologies have not been clearly defined. Exposure to metals is however, associated with increased levels of intracellular oxidative damage, including lipid peroxidation, protein denaturation, and DNA strand breaks [4]. Increased oxidative stress may be caused by metal-catalyzed redox reactions, depletion of glutathione, or inhibition of the enzymes that remove reactive oxygen species [4]. Transition metals can also alter the function of proteins by directly binding to sulfhydryl groups or by substituting for metal cofactors [5]. In addition to cellular damage, exposure to metals is associated with the activation of a variety of intracellular signal transduction pathways including those regulated by mitogen activated protein kinases (MAPKs), NF-κB, and calcium-dependant kinases [4]. The inappropriate activation of these pathways may contribute to the etiology of metal-induced cancer and developmental abnormalities (reviewed in: [3], [5]–[9]).

To defend against metal toxicity, sophisticated defense mechanisms have evolved. These include regulating intracellular metal concentrations via chelation and ion pumps, removal of reactive oxygen species, and repair of metal-induced damage [8]. Although many of the genes and cognate regulatory pathways have been identified, the consequence of metal exposure on a systematic level has not been examined. Several questions remain including: (a) what are the global effects of metal exposure on the eukaryotic transcriptome; (b) what are the similarities/differences in the transcriptional response among transition metals and metalloids; (c) what are the roles of differentially expressed genes in the defense against metal toxicity; and (d) are there additional metal-responsive genes and regulatory pathways?

To address these gaps in our understanding of the molecular biology of transition metals, the genomic effects of exposure to Group IB (copper, silver), IIB (zinc, cadmium, mercury), VIA (chromium) and VB (arsenic) elements on the yeast *Saccharomyces cerevisiae* were examined. Mechanisms of metal homeostasis and tolerance have been examined in *Saccharomyces cerevisiae* [8]. Like other eukaryotes, yeast respond to metals by decreasing metal accumulation, increasing metal chelation, and compartmentalizing metal-ligand complexes 10–12. In this report, two comprehensive sets of metal-responsive genomic profiles are presented: one that provides information on the transcriptional changes associated with metal exposure (transcriptome), and a second that provides information on the relationship between the expression of ∼4,700 non-essential genes and sensitivity to metal exposure (deletome). These global genomic profiles were integrated to identify cellular pathways that regulate gene expression and are required for cell survival under toxic conditions. Integrating genomic profiles for gene expression and growth phenotype is a powerful tool for understanding the mechanisms involved in global responses and adaptation [13],[14]. To properly integrate the responses, transcriptomes, after exposure to equi-toxic concentrations of different metals/metalloids, were examined using a single strain of wild type, diploid yeast cultured under consistent growth conditions, DNA microarray platforms, data extraction, and analysis protocols. Thus, differences in the genomic responses can be attributed to the effects of different chemical exposures.

## Results

### Metal-Responsive Transcriptome

A total of 1,341 unique genes were differentially expressed among the fourteen exposure conditions (seven metals at two concentrations). Arsenic (1.25 mM) affected the expression of the largest number of genes, 762; while cadmium (25 µM) affected the fewest, 174. The majority of the treatments changed the expression of 230 to 470 genes (Table 1).

**Table 1:** Summary of Differentially Expressed Genes and Metal-Sensitive strains.

Metal	Concentration (µM)	Responsive Genes	
		Gene Expression	Growth Inhibition^a^
Silver	10	232	0
	20	319	3
Copper	5000	247	4
	7000		108
	9000	467	
Cadmium	5	180	24
	25	174	256
Mercury	19	302	0
	47	233	5
Zinc	1000	329	24
	2000	404	87
Chromium	400	279	64
	900		209
	1700	227	
Arsenic	400	381	5
	1250	762	65

a Number of strains whose gene deletion causes a >50% reduction in growth in the presence of metal, relative to the metal-treated, control yeast strain.

**Figure 1:**
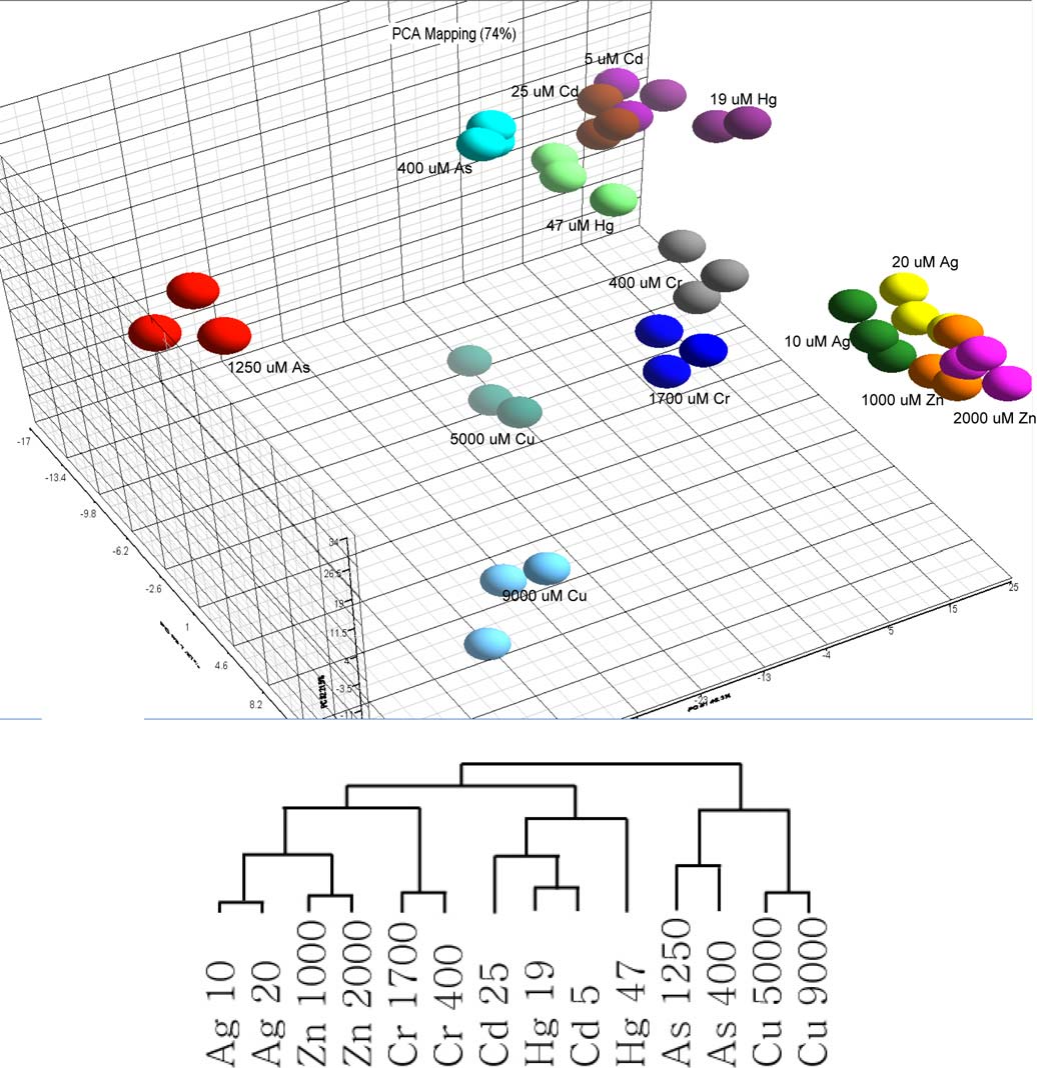
Principal Component and hierarchical cluster analyses. The fold-change of 1,341 differentially expressed genes was used in these analyses. (*Upper Panel*) Principal Component Analyses of three pairs of independent biological replicates with the same treatments are designated with identical colors. (*Lower Panel*) Hierarchical cluster by treatment of the gene expression data. This hierarchical cluster was calculated using the unfiltered 87,304 gene-treatment expression dataset.

Principal Component Analysis (PCA) showed tight clustering of the three biological replicates for each treatment, indicating the reproducibility of the experiments (Figure 1). Expression patterns associated with the two concentrations of the same metal were sufficiently different to distribute the samples into separate but adjacent clusters, suggesting similarities in their transcription profiles. PCA also suggested that the type of metal or its chemical/toxicological profile was a major contributing factor in the transcriptional profile. For example, cadmium and mercury, which are mammalian toxicants and Group IIB metals, formed a separate, tight cluster. Chromium is unique in that it is a Group VIB metal, while the other metals are Groups IB or IIB. Both concentrations of chromium clustered together, but were separate from the other metals. Copper was also separate from the other IB and IIB metals. Copper is unique in that it is the only IB metal that is redox active *in vivo*. Arsenic is also redox active *in vivo* and clustered close to copper. The highest concentration of arsenic (1.25 mM) was separate from the other treatments. Hierarchical clustering by treatment showed a similar grouping of metals: the redox active elements copper and arsenic clustered together separate from the other metals. Likewise, chromium was separate from the other metals (Figure 1).

### Common Metal Responsive (CMR) Genes

**Figure 2:**
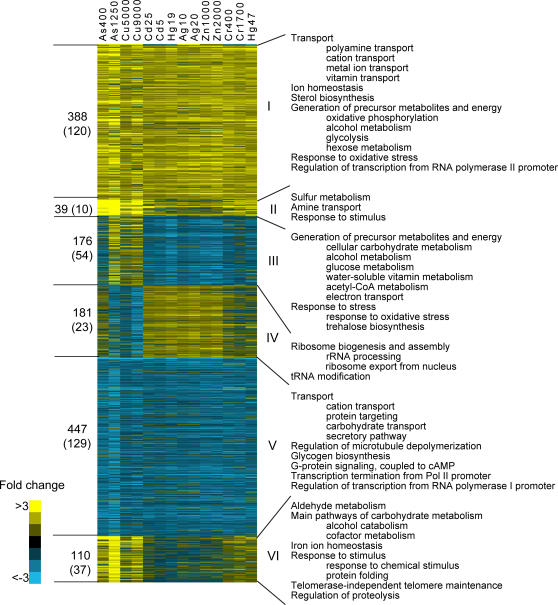
K-means cluster of differentially expressed genes and selected enriched Gene Ontology terms. K-means clustering on the average fold-change in gene expression values was performed with K = 6 for the genes, K = 3 for the samples Euclidean distance as the similarity metric. Metal species with concentration values (µM) are presented at the top of each column. The values on the left side of the heat map denote the number of genes in each cluster, with the values in the parenthesis indicating the number of genes with unknown Gene Ontologies. Expression values, measures of significance, and corresponding Gene Ontologies for the genes and clusters presented in this Figure can be found in Tables S1 and S2, respectively. The hierarchical cluster presented at the top of the heat-map was calculated using the 1,341 differentially expressed genes dataset used in the K-means clustering analysis.

K-means clustering identified six distinct groups of genes (Figure 2, Table S1). The two largest clusters, Cluster I (induced genes) and Cluster V (repressed genes) contained 388 and 447 genes, respectively. The expression of these genes was similarly affected by all metals; therefore they are referred to as common metal responsive (CMR) genes.

### Cluster I - Induced CMR Genes

Significantly enriched biological processes for the induced-CMR genes included metal ion transport and homeostasis, detoxification of reactive oxygen species, carbohydrate metabolism, fatty acid metabolism, polyamine transport, and RNA polymerase II transcription (Figure 2, Table S2). Several of these gene products are involved in glycolysis, oxidative phosphorylation and alcohol metabolism, and are necessary for energy production of ATP-dependent molecular chaperons and other cellular stress responses [15]. Genes involved in response to reactive oxygen species were also induced, which was expected since transition metal exposure is associated with increased oxidative stress [4]. The expression of polyamine transporter genes *TPO3, TPO1, TPO2,* and *PTK1* was induced. Polyamines also protect yeast from reactive oxygen species [16]. The expression of genes involved in maintaining ion homeostasis (*NFS1, SOD1, ATX1, FRE8, SMF3, CCC2, ATM1*) and iron transport (*ARN1, ARN2, SIT1, FTR1, FET3*) were also induced. Increased expression of these genes may serve a protective role by removing excessive metals. It may also reflect a disruption in normal metal metabolism. For example, *ARN1, ARN2, SIT1, FTR1,* and *FET3* are normally regulated by the iron transcription factors, Aft1 and Aft2, which are activated when iron is scarce [17],[18]. Thus, exposure to elevated levels of one metal may disrupt the metal sensing systems of others.

### Cluster V - Repressed CMR Genes

The significantly enriched biological processes of repressed CMR genes included polysaccharide biosynthesis, G-protein signaling, protein targeting, and transport (Figure 2, Table S2). Genes encoding subunits of protein kinase CK2 (*CKB2*, *CKA1*, *CKA2*) were repressed, suggesting that metal exposure could lead to the inhibition of protein kinase CK2 responsive signaling. The expression of transition metal transporters, *CTR1*, *FET4*, *LPE10*, *BSD2*, *COX17*, *ZRT1* and *ZRT2* was repressed, which could result in a decrease in metal uptake. Interestingly, genes encoding carbohydrate transporters (*HXT2, HXT4, HXT6, HXT7, MTH1, MAL11, MAL31*) were also repressed. It is known that hexose transporters transport arsenite [8],[19]. Thus, the suppression of these transporters may lead to a decrease in intracellular metal concentrations.

### Other Clusters

The differences in Clusters II, III, IV and VI can be partially attributed to the expression patterns associated with copper and arsenic (Figure 2). Cluster III shows increased gene expression in cells exposed to the highest concentration of arsenic and both concentrations of copper, and repression with other metals. The biological processes enriched in this cluster include energy generation, response to stress, and trehalose biosynthesis (Figure 2, Table S2). These processes are similar to those identified in Cluster I. Cluster IV showed an opposite pattern of expression compared to Cluster III. Enriched biological processes in Cluster IV were related to ribosome biogenesis and assembly. Some of the genes in this cluster encode ribosomal proteins; however, many are components of large or small ribosomal subunit processomes. Processomes are ribonucleoproteins required for the processing of 35S primary transcripts, 20S pre-rRNAs and 27S pre-RNAs [20]. Genes involved in transcription termination (*RTT103*, *SYC1*, *RAI1 SWD2*) were also repressed. These proteins are responsible for the generation of the free 3′ end of mRNA and are involved in the transcription termination of RNA polymerase II after passing the poly(A) site [21]. Cluster II contains genes involved in the biological processes of sulfur metabolism and response to oxidative stress. Arsenic, mercury, chromium and cadmium induced the expression of genes that function in sulphate assimilation and glutathione biosynthesis pathways [14], [22]–[24]. Enriched biological processes in Cluster VI included protein folding, telomere maintenance, and proteolysis.

### Interactomes

Cytoscape was used to identify the metal-responsive protein-protein and protein-DNA interacting networks [25],[26]. The ten most significant sub-networks are presented in Table 2. The majority of the biological processes associated with the genes at the center of the sub-networks for the Group IB and IIB transition metals were involved in small and large ribosomal subunit processomes [27],[28]: silver (10/10), cadmium (10/10), copper (9/10), mercury (7/10) and zinc (6/10). The sub-networks for chromium were unique and centered on transcription factors, proteasomes and kinases. The sub-networks for the metalloid arsenic were also unique with nine of the sub-networks centered on transcription factors involved in stress tolerance. Both arsenic and copper contain the sub-network centered around the stress activated transcription factor MSN2 [29].

**Table 2:** Ten Most Significant Interacting Sub-networks.

Rank^a^	Metal						
	Silver	Arsenic	Cadmium	Chromium	Copper	Mercury	Zinc
1	**PWP2**	*YAP7*	**PWP2**	*DIG1*	**NOP2**	**PWP2**	**PWP2**
2	**NOP1**	*HSF1*	**UTP18**	***PKH2***	**ERB1**	**UTP7**	**NOP1**
3	**KRR1**	*MSN2*	**UTP7**	*MET4*	**NOP4**	PRE1	**UTP7**
4	**NOP15**	*MSN4*	**KRR1**	RPN6	**MAK21**	**UTP22**	**NOP6**
5	**UTP18**	*YAP1*	**ENP1**	CRG1	**SSF1**	**NOP2**	RPN3
6	**UTP7**	*MET32*	**NOP1**	HHT1	**RPF2**	**UTP18**	***PKH2***
7	**NOP4**	*CAD1*	**NOP6**	MAL32	**NOP15**	***PKH2***	RPN11
8	**NOP6**	*MET4*	**UTP22**	IRA1	*MSN2*	**UTP15**	**NOP15**
9	**NOP7**	*PST1*	**NOP15**	***CKS1***	**MAK5**	**AAH1**	**REI1**
10	**ENP1**	***ATG17***	**DIP2**	**NOC3**	**NOP6**	RPN3	EHD3

a Rank is based on level of significance, with Z-score >2. Genes are highlighted based on biological processes: **bold**, rRNA processing and ribosome assembly; *italic*, transcription factors; ***bold-italic***, kinase; and underline, proteasome. Genes that are not highlighted are unique or have not been assigned GO categories (HHT1, chromatin assembly; MAL32, maltose catabolism; IRA1, intracellular signal cascade; EHD3, vesicle-mediated transport; CRG1, unassigned).

### Deletome

To identify genes whose deletion renders yeast most sensitive to metal toxicity, a GIF≥5 (≥80% growth inhibition) was applied to the deletome of yeast exposed to metals at EC_10_ (Table S3). Forty two genes were identified whose deletion caused a significant growth inhibition at the EC_10_. Genes involved in vacuole organization and biogenesis were essential for viability at low chromium and zinc concentrations (Table 3). Genes involved in the maintenance of cell wall integrity and those needed for bud neck formation mediated resistance to cadmium, chromium or copper toxicity. Protein kinase mutants (*slt2Δ, bck1Δ, ypk1Δ*) are also very sensitive to cadmium and chromium treatments. *SLT2* and *BCK1* encode MAPK and MAPKKK, respectively, which are regulated by *PKC1*-mediated signaling pathways [30]. Mutants *cys3Δ*, *cys4Δ*, (cysteine biosynthesis) *sam1Δ* (S-adenosylmethionine synthesis), and *sod1Δ* (reactive oxygen scavenging) were sensitive to chromium. These results suggest that maintaining cell wall integrity, chelating metals, sequestering metals in vacuoles, and reducing oxidative stress are fundamental processes for mediating resistance to metal toxicity.

**Table 3:** Genes that are Essential for Resistance to Metal Toxicity.

Metal	Genes/ORF Deletion^a^	Gene Ontology - Biological Process
**Arsenic**	*ADH1*	Alcohol dehydrogenase
	*YML095C-A*	Unknown
**Cadmium**	*SLT2, BCK1*	Maintenance of cell wall integrity; MAP kinase pathway regulated by the PKC1-mediated signaling pathway
	*CYC8*	General transcriptional co-repressor/co-activator
	*BUD25*	Protein involved in bud-site selection
**Chromium**	*CUP5, PEP3, PPA1, VMA10/YHR039C-B, VMA13, VMA2, VMA21, VMA22, VMA4, VMA6, VMA7, VMA8, VMA9, VPH2, VPS16, VPS34*	Vacuole organization and biogenesis
	*CYS3, CYS4, SAM1*	Sulphur amino acid metabolism
	*SOD1*	Cytosolic superoxide dismutase
	*BUD16*	Protein involved in bud-site selection and telomere maintenance
	*YPK1, SLT2*	Maintenance cell integrity signaling pathways; of Serine/threonine protein kinase
	*IMP2′, NPR2, TPD3*	Transcriptional regulators
	*MDM20*	Mitochondrial inheritance and actin assembly
	*VRP1*	Cytoskeletal organization and cytokinesis
	*RMD11, YNL140C, YOR331C*	Unknown
**Copper**	*CUP2, YGL165C (Dubious, partially overlaps CUP2)*	Copper-binding transcription factor
	*OCH1*	Mannosyltransferase
**Zinc**	*VMA10, PKR1, CUP5, PEP3, PPA1, VMA13, VMA2, VMA21, VMA22, VMA4, VMA5, VMA6, VMA7, VMA8, VMA9, VPH2, VPS33, VPS34*	Vacuole organization and biogenesis
**Mercury**		
**Silver**		

a Mutants showing 80% growth inhibition at EC_10_ metal concentrations.

**Figure 3:**
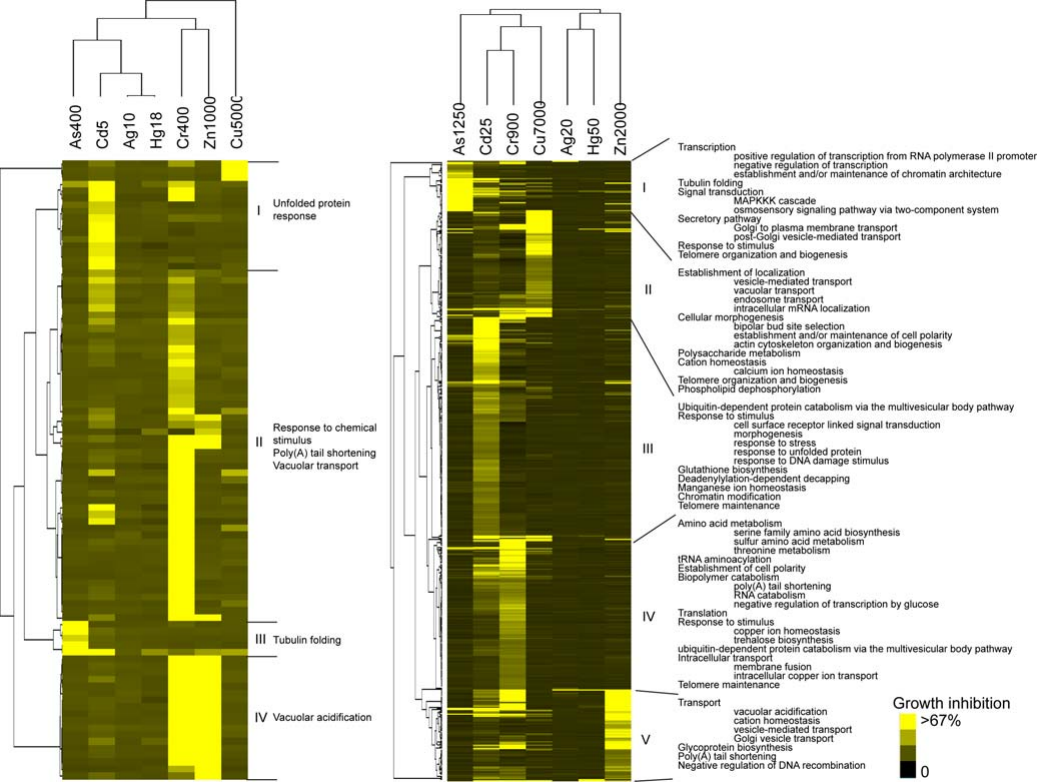
Two-dimensional hierarchical cluster of GIF's for EC_10_ (*left panel*) or EC_50_ (*right panel*) deletomes. The average linkage clustering method and Pearson correlation (uncentered) as a similarity measure were used to group the samples and the genes. The metal concentrations (µM) are shown at the top of each column. Selected Gene Ontology terms that are highly enriched in each cluster were shown. GIF's and corresponding Gene Ontologies for the genes presented in this Figure can be found in Tables S3 and S4, respectively.

Using a GIF≥2, 90 and 540 strains were identified that showed growth inhibition at the EC_10_ and EC_50_, respectively (Figure 3, Table S3). 98% of the mutants that were sensitive at the EC_10_ were also sensitive at the EC_50_. Silver and mercury affected the growth of the fewest number of deletion strains, while cadmium and chromium affected the highest number of strains (Table 1).

Hierarchical cluster analysis revealed that the type of metal defined the genes contained within each cluster in the deletome (Figure 3). This is in contrast to the transcriptome results in which clusters contained genes responding to all (e.g., CMR) or several metals. This suggests that resistance to metal toxicity involves metal specific responses, while the effect on transcription may be partially metal-independent. Gene Ontology analysis of the genes in the five major clusters for the deletome indicates that Cluster I, which is dominated by mutants sensitive to arsenic, was significantly enriched in the biological processes of stress-related transcription regulation, tubulin folding, signal transduction, secretory pathway, and response to stimulus (Figure 3, Table S4). Cluster II, which is dominated by mutants sensitive to copper, was enriched in biological processes of vesicle mediated transport mechanism. Mutants for cellular morphogenesis (*sac6Δ, end3Δ, cln3Δ, bni1Δ, tpm1Δ*) and cation homeostasis (*trk1Δ, hal5Δ, csg2Δ, nhx1Δ, sat4Δ, spf1Δ*) were also sensitive to copper. Enriched biological processes that map to Cluster III, which is dominated by mutants sensitive to cadmium, contained cell surface receptor-linked signal transduction, morphogenesis, chromatin modification, glutathione biosynthesis, and response to stress; including DNA damage. Sulfur amino acid metabolism, ubiquitin-dependent protein sorting, and trehalose biosynthesis were enriched in Cluster IV, which is dominated by mutants sensitive to chromium. Cluster V, which is dominated by mutants sensitive to zinc, was enriched with vesicle-mediated transport processes, as well as poly (A) tail shortening and negative regulation of DNA recombination.

**Figure 4:**
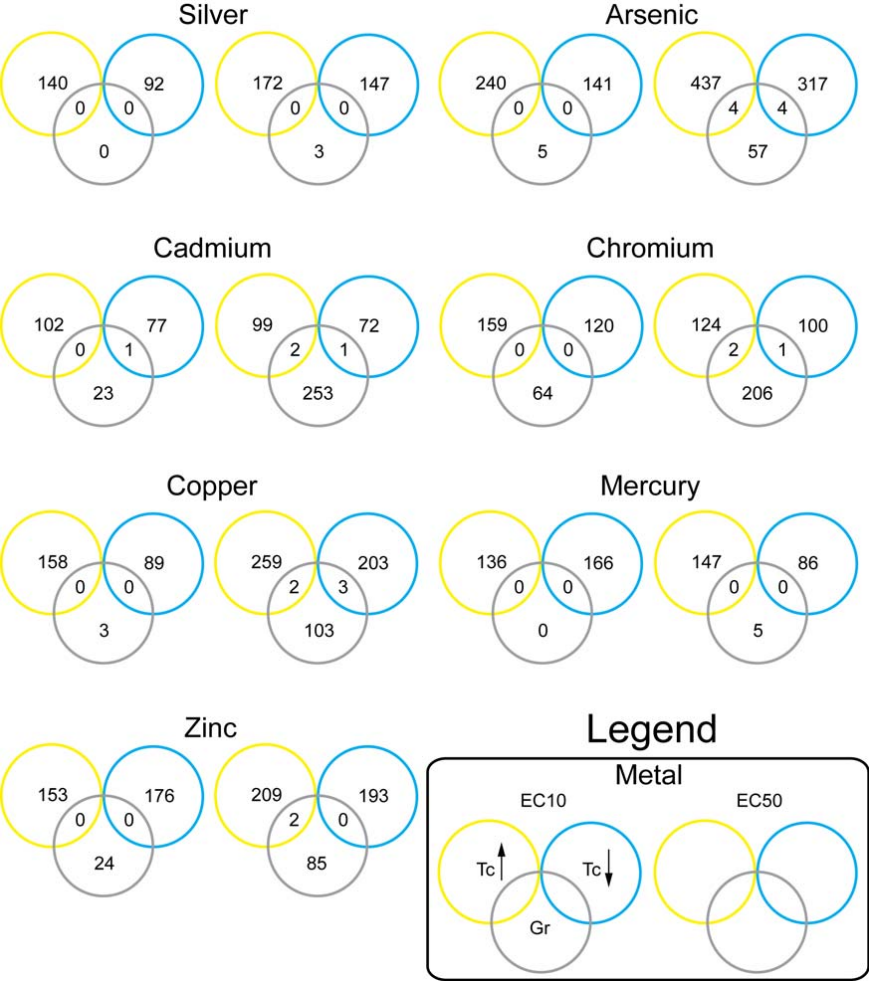
Identification of common genes between the metal-responsive transcriptome and deletome. Venn diagrams illustrate the distribution of genes whose level of expression increased (*Tc*↑), decreased (*Tc*↓) and/or that were essential for resistance to metal toxicity (*Gr*). Genes located in the intersections of the datasets and their ontologies are presented in Table S5.

When similar treatment conditions were compared, less than 2% of the genes identified in the metal-responsive transcriptome were found to affect resistance to metal toxicity (Figure 4). At a GIF≥2, eight genes were required for resistance to silver and mercury toxicity. However, exposure to identical concentrations of silver and mercury resulted in the differential expression of 551 and 533 genes respectively (Figure 4, Table 1). Cadmium at EC_50_ affected the transcription of the lowest number of genes; however, it caused growth inhibition in the highest number of mutant yeast stains. Overlapping regions in the transcriptome/deletome Venn diagrams identified *CYS3* as both transcriptionally responsive and essential for viability in the presence of arsenic, cadmium and copper. Similarly, *ADH1* was required for resistance to arsenic and copper toxicity, and its expression was up-regulated by these metals (Table S5).

**Figure 5:**
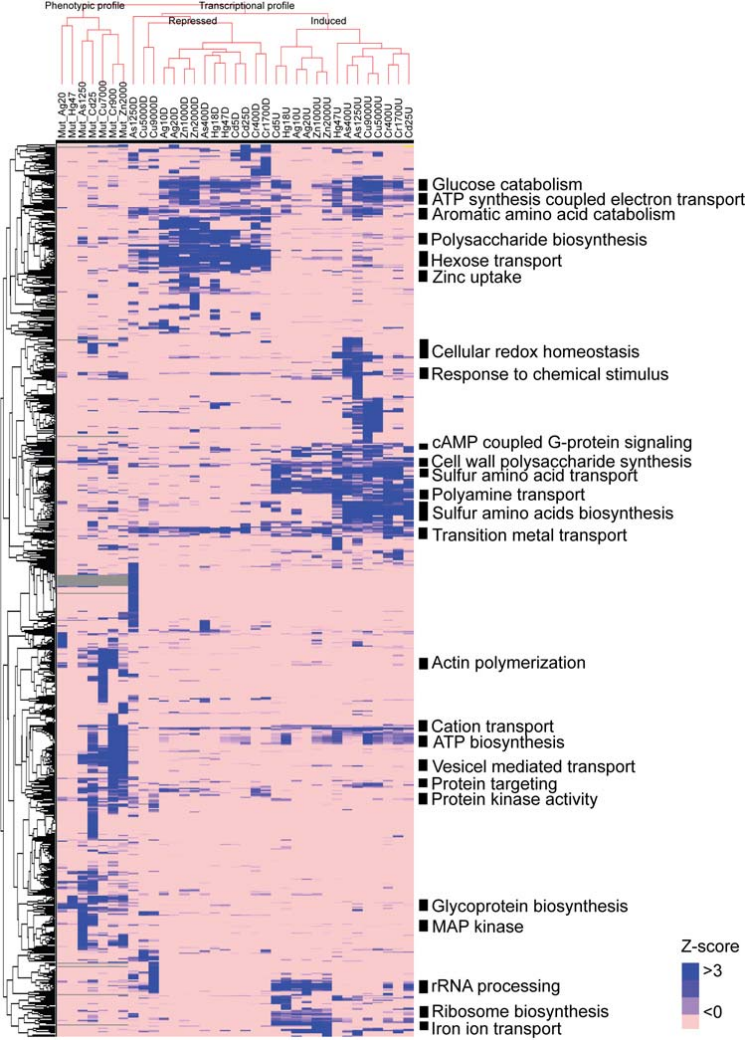
Two dimensional hierarchical clustering of transcriptome and deletome Gene Ontology terms. Clustering of the data is as described in the legend to Figure 3. Metal species and concentrations (µM) are indicated at the top of each column. Labels with the *Mut* prefix indicate deletome data. Labels containing a *U* or *D* suffix indicate transcriptome data for genes whose level of expression increased or decreased in response to metal exposure, respectively. The Gene Ontology Z-score data presented in this Figure can be found in Table S6.

The results from the Gene Ontology analyses of transcriptome and deletome profiles were combined and a matrix was constructed. Z-scores for each GO term were combined, clustered and visualized (Figure 5, Table S6). Hierarchical clustering by experiment resulted in three distinctive clusters: growth inhibition screening (i.e., deletome), down-regulated genes, and up-regulated genes. The biological processes of cation and transition metal transports were common among the three experimental groups. Sulfur amino acid transport and biosynthesis, enriched in the up-regulated gene cluster, was also required for cell growth under arsenic, cadmium, chromium and copper treatments (Figure 5).

## Discussion

Global transcriptome and deletome profiles for yeast exposed to Group IB (copper, silver), IIB (zinc, cadmium, mercury), VIA (chromium) and VB (arsenic) elements were generated and compared. Genomic data sets were generated using an experimental design to minimize variations associated with differences in yeast strain backgrounds, microarray platforms, and data extraction and analysis techniques. In addition, equi-toxic concentrations of metals were used to minimize differences due to variations in the level of toxicity. Several studies have shown that minimizing experimental variation is essential for decreasing statistical variability in microarray data [31],[32]. Thus, by minimizing experimental variations, transcriptional responses associated primarily with the different metals were identified.

Several studies have compared the effects of exposure to multiple metals on the transcriptome in yeast and mammalian cells. There was, however, little overlap between the responsive genes reported in these studies and those presented in the current report. When enriched GO categories were compared however, genes involved in detoxification of oxidative stress commonly respond to stress conditions [33]–[35].

Transcriptome analysis revealed that each of the seven metals affected the level of expression of 175–760 genes (Table 1). PCA and hierarchical clustering by experimental conditions grouped the expression patterns by physiological/chemical characteristics of the metals. PCA and clustering placed cadmium and mercury, two non-essential metals, close together and separate from the essential metals; zinc, chromium, and copper. Silver closely grouped with zinc. This may be because silver can substitute for other metals in many biological activities in yeast and may be sensed as an essential metal [36],[37]. Although chromium is essential, it is a Group VIB transition metal with different chemical properties compared to the others. These results suggest that the chemical properties of the metal/metalloid contribute to the pattern of the genomic response.

K-means clustering illustrated that the expression of ∼60% of the genes was similarly affected by all seven metals. In addition, two sub-sets of metals: (A) zinc, chromium, mercury, silver and cadmium; and (B) arsenic and copper, affected the expression of similar genes. This suggests that cells may have developed common or convergent transcription regulatory mechanisms to accommodate metal exposure. Copper and arsenic, which undergo redox reactions *in vivo*, affected the expression of similar genes. This suggests that the ability to participate in redox reactions defines a unique set of metal-responsive genes. Although chromium is redox active *in vivo*, it clustered distinctly from copper and arsenic (Figure 2). Several chromium responsive genes in Clusters IV and VI did, however, show expression levels similar to those of copper and arsenic. These results suggest that the ability to undergo redox reactions *in vivo* contributes to the global transcriptional response, but other chemical properties of the metal may dominate the response.

Generating precursor metabolites and energy, synthesizing proteins and amino acids, transporting chemicals and proteins, and responding to oxidative stress were common responses to metal exposure. Furthermore, there were increases in detoxifying processes such as metal chelation, sequestration of metals into endosomal vacuoles, and exocytosis. These responses may reduce the intracellular levels of the metal by decreasing the number of metal-importing transporters and increasing metal efflux through major facilitators. The processes identified in this study are similar to adaptations in yeast exposed to other environmental stressors, as well as in human fibroblasts exposed to arsenic [15], [38]–[40].

The expression of genes associated with the production of ribosomes was also affected by metal exposure. Other environmental stresses have also been shown to repress ribosomal protein expression and the translation apparatus [38],[41],[42]. Ribosome production may utilize more than 50% of the synthetic effort of rapidly growing eukaryotic cells [41],[43]. Therefore, by inhibiting ribosome synthesis, cells may be able to redirect these resources towards the defense against metal toxicity [44].

Analyses of the transcriptome and deletome identified several evolutionarily conserved signal transduction pathways that may be involved in regulating the responses to metal exposure. These include those mediated by cAMP-dependent protein kinase A (PKA), protein kinase CK2, and MAPK.

The PKA pathway coordinates post-translational regulation of a variety of proteins such as key enzymes of glycolysis and gluconeogenesis, and the transcriptional control of ribosomal protein and stress response proteins [45]–[47]. Of the 1,341 metal responsive genes ∼10% are stress response genes whose expression is controlled by the PKA-regulated transcription factors Msn2p/Msn4p [29],[48],[49]. When PKA is activated, Msn2p/Msn4p activity is repressed. Non-activation of PKA due to low cAMP levels will ultimately lead to the derepression of Msn2p/Msn4p [50]. The mechanism by which metals inactivate PKA has not been fully defined; however, our results suggest that metal exposure may reduce the levels of cytoplasmic glucose. In yeast, low glucose causes a decrease in the level of cAMP with concomitant low PKA activity [51]–[55]. Changes in glucose levels may be the result of a combination of changes in yeast metabolism. There were decreases in the expression of high affinity hexose plasma membrane transporters (*HXT2, HXT4, HXT6, HXT7*) and increases in the expression of genes associated with glycolysis and hexose metabolism (*PGK1 ENO2 FBA1 TYE7/SGC1 CDC19*) (Figure 2; Table S1). Furthermore, there was a decrease in the expression of the maltase genes *MAL12* and *MAL32*, which metabolize maltose into glucose [56],[57]. The combination of decreased sugar production and transport, and increased metabolism may lead to a level of glucose that would limit PKA activity. This will ultimately lead to increased stress response gene expression via Msn2p/Msn4p.

Protein kinase CK2 functions in diverse cellular processes [58],[59]. Protein kinase CK2, along with Utp22, Rrp7 and Ifh1 comprises the CURI protein complex. It has been suggested that the CURI complex is involved in ribosome synthesis, mediating transcription and processing of pre-rRNAs, and the transcription of ribosomal protein genes [60]. Metal treatment caused a decrease in *CKA1*, *CKA2* and *CKB2* expression. This is similar to the results obtained in arsenic exposed JB6 mouse epidermal cells [61]. Thus, decreased ribosome biosynthesis associated with metal exposure may be caused by the repression of protein kinase CK2, as well as decreased PKA activity [14],[55],[62].

MAPK cascades participate in various cellular processes including apoptosis, differentiation and the stress response. MAPKs have been shown to mediate inducible transcription in response to exposure of the seven metals examined in this study [63]–[65]. In yeast, five MAPKs have been identified [66]. Among them, *HOG1*, a mammalian p38 homolog, regulates the high osmolarity glycerol response, which is activated through two independent upstream pathways that converge at MAPKK *PBS2* [67]–[69]. The deletome analysis revealed that proteins in the high osmolarity glycerol response (Sho1, Ste20, Ssk1, Ssk2, Pbs2, Hog1) are required for arsenic, cadmium and zinc tolerance. Another MAPK cascade that regulates the cell wall construction pathway (Bck1, Mkk1) was required for cadmium tolerance. Cytoscape analysis of the transcriptome revealed that a group of proteins that interact with the MAPKKK Pkh2, a serine/threonine kinase involved in maintenance of cell wall integrity, significantly changed in their levels of expression (Table 2). These results are consistent with other studies that demonstrated a role of MAPK cascades in controlling metal-responsive transcription. Furthermore, they indicate that proper functioning of the MAPK pathways is essential for survival to environmental stresses.

The deletome was created by measuring the growth characteristics of each mutant strain in the presence of metal. Hierarchical clustering showed limited overlap in the genes comprising the different clusters of mutants exposed to the metals (Figure 3). This suggests that resistance to metal toxicity may have divergent mechanisms.

Mercury and silver had the lowest number of genes in the deletome. This may be a consequence in how yeast responds to exposure of these metals. When yeast was grown in increasing concentrations of these metals, there was a concentration-dependent increase in the length of the stationary growth phases. However, exponential growth rates and maximal cell densities were similar at almost all of the mercury and silver concentrations (Figure S1). In contrast, growth in the presence of the other metals caused concentration-dependent decreases in growth rates and maximal cell densities. Thus, yeast may better adapt to silver and mercury exposure. Exposure to metals at concentrations beyond which yeast can adapt may reveal additional genes in the deletome. Alternatively, monitoring deletion strains for metal-induced changes in the stationary phase identify other essential genes.

A comparison of genes in the transcriptome and deletome did not identify many common genes (Figure 4). This lack of correlation has been observed in other studies. It may be attributed to genetic redundancy or the inability of microarrays to measure non-transcriptionally regulated changes in activity [13],[14],[70],[71]. The lack of overlap may be due to differences between the measured end points. For the transcriptome, RNA was purified from the cells exposed to metals for 2 hours, during lag phase of growth. Yeast begin to respond to new environments and adapt for growth during this period of time. Thus, the genes identified in the transcriptome may contribute to the early adaptation of metal-induced stress. In contrast, GIF's were calculated from yeast in stationary growth phase and may be related to cell proliferation. Many of the other yeast deletomes also examined cells that are in the stationary phase [70],[72],[73]. In the future, a greater overlap between the transcriptome and deletome may be achieved if GIF's are calculated from metal-induced differences in lag times, initial growth rates, and maximum cell density.

By combining Gene Ontology results from the transcriptome and deletome, several common processes were identified (Figure 5). These include cation and transition metal transport, and sulfur amino acid transport and biosynthesis, which were required for cell growth in the presence of arsenic, cadmium, chromium and copper. Gene Ontology results suggest that genes in the deletome may represent convergent or central upstream points in pathways, which ultimately protect cells against metal toxicity. Furthermore, the transcriptome may identify the downstream genes in the cognate pathways. For example, the deletome contained genes involved in serine and threonine metabolism; glutamate, aspartate and arginine metabolism; and shikimate metabolism. These genes are located upstream in the sulfur, methionine and homocysteine metabolic pathways. The downstream genes in these pathways were identified in the transcriptome. Changes in sulfur amino acids is consistent with results obtained in previous studies of arsenic exposure, where processes involved in glutathione synthesis overlapped in the transcriptome and deletome [14].

The capacity to respond to environmental stresses is critical to the survival and propagation of all organisms. Genomic studies provide important information on molecular mechanisms of environmental stress responses. In this study, we have identified genes that respond to exposure of essential and non-essential metals. In addition, genes that are essential for survival in the presence of these metals were identified. This information will contribute to our understanding of the molecular mechanisms by which organisms respond to metal stress, and could lead to an understanding of the connection between environmental stress and signal transduction pathways.

## Materials and Methods

### Strains, Media, and Growth Conditions

The homozygous diploid *Saccharomyces cerevisiae* strain BY4743, the result of mating of two haploid strains BY4741 (*MATa his3Δ1 leu2Δ0 met15Δ0 ura3Δ0*) and BY4742 (*MATα his3Δ1 leu2Δ0 lys2Δ0 ura3Δ0*) [74], was used for all transcriptome and deletome studies. Yeast was grown in YPD (1% yeast extract, 2% bactopeptone, 2% glucose) at 30°C.

### Cytotoxicity Assays

To define equi-toxic concentrations of the metals, the effects of different concentrations of AgNO_3_, NaAsO_2_, CdCl_2_, CrO_3_, CuSO_4_, HgCl_2_, and ZnSO_4_ on cell growth and viability were determined. Cell growth was measured by first diluting an overnight culture of yeast to an optical density of 0.3, at 600 nm, in YPD medium, and then incubating for 3 h. This culture was then diluted to an optical density of 0.01 with YPD containing various concentrations of metal. Yeast were incubated at 30°C, and growth was monitored by observing changes in OD_600_ as a function of time (Figure S1). The EC_10_ and EC_50_, concentration of added metal that inhibits yeast growth by 10% and 50%, respectively, for each metal were then calculated using Toxstat ver. 3.4 (WEST, Inc., Cheyenne, WY).

The effect of metal exposure on cell viability was assessed by counting the number of viable yeast cells remaining following 1, 2 and 3 h exposures of log phase cells to different concentrations of metal (Figure S1). The EC_10_ and EC_50_ concentrations that were used in subsequent transcriptome and deletome studies did not have a significant effect on cell viability (Figure S1).

### RNA Purification and Microarray Hybridization

To prepare RNA for microarray studies, three independent colonies of yeast were grown overnight in YPD. These cultures were then diluted 1∶2.3 with YPD, and incubated for an additional 3 h at 30°C. Cells were then diluted to optical density of 0.1 (∼10^6^ cells/ml) in YPD containing metals at either the EC_10_ or EC_50_ (Table 4), or no metal added YPD. Yeast were harvested following a 2 h incubation at 30°C, and total RNA was purified as previously described [75]. The quality of the purified RNA was determined with a BioAnalyzer (Agilent Technologies, Palo Alto, CA), and the RNA was then stored at −70°C.

**Table 4:** Concentrations of metals used in microarray and deletion-strain growth studies.

Metal	Concentration (µM)	
	EC_10_	EC_50_
silver	10	20
arsenic	400	1250
cadmium	5	25
chromium^a^	400	1700
copper^a^	5000	9000
mercury	19	47
zinc	1000	2000

a For the deletome, the EC_50_ copper and chromium concentrations were reduced from 9000 µM to 7000 µM and from 1700 to 900 µM, respectively.

For microarray hybridizations, 100 ng of total RNA from metal-treated yeast was amplified and labeled with Cy3 fluorescent dye, and a common reference pool (no metal added control) was amplified and labeled with Cy5 using Agilent Technologies Low RNA Input Linear Amplification labeling kit following the manufacturer's protocol. Equal amounts of Cy3- and Cy5-lableled cRNA were then hybridized to an Agilent Yeast Oligonucleotide microarray (Cat. No. G4140B) for 17 h at 65°C. The hybridized microarrays were then washed and scanned using an Agilent G2565BA scanner. Data were extracted using Agilent's Feature Extraction software. A total of 84 microarrays were analyzed in this study: 7 metals×2 concentrations×3 biological replicates×2 (dye-swap). This results in six measurements for each treatment condition.

### Deletome Measurements

To define the deletome, the effect of each metal on the growth of individual yeast strains in a homozygous diploid gene deletion library (Open Biosystems Huntsville, AL, Cat. No. YSC1056) was determined. For growth measurements, a 5 µl sample of each strain was transferred into 195 µl of fresh YPD, in each well of a 96-well plate. For a normalization control, about 10^3^ cells of wild type BY4743 were inoculated into each plate. After 2 days of standing incubation at 30°C, the plate was replicated into thirty 96-well plates containing YPD. Each replicate contained one of the seven metals at one of two concentrations (Table 4), as well as one replicate with no added metal. There were two replicates for each of the fifteen different experimental conditions to yield a total of thirty plates from each original library plate. Cells were maintained as standing cultures at room temperature, and the optical density at 620 nm was measured for each replicate plate every 90 min for 42 h using a Biomek® FX Laboratory Automation Workstation equipped with a DTX800 Multimode Detector (Beckman Coulter, Fullerton, CA). This process was repeated for each of the 56 plates in the original library, resulting in two replicate growth curves for each of the 4,739 genes at 15 different metal exposure conditions.

### Growth Inhibition Profiling

For each deletion strain, treatment and replicate, the minimum OD_620_ was subtracted from the maximum OD_620_ over the 42 h period to estimate cell growth. These growth estimates were used to identify strains with metal-responsive genes as follows: Let *g_s,m_* be the estimated change in OD for a strain with gene deletion *s* (*s* = 1,…,4739) and metal treatment *m* (*m* = 1,…,15), where *s* = 1 indicates the strain without a deleted gene, and *m* = 1 indicates the absence of metal. Then for each strain with a deleted gene, a growth inhibition factor (GIF) was calculated as the ratio of the growth of the strain with deleted gene *s* in the absence of metal (g_s,1_) as a fraction of the growth of the wild type strain in the absence of metal (g_1,1_) to the growth of strain with deleted gene *s* for metal treatment *m* (g_s,m_)as a fraction of the growth of the wild type strain for the same metal treatment *m* (g_1,m_). That is,
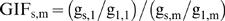



Larger values of the GIF indicate greater growth inhibition. Deletions that significantly affect growth in the presence of metal are defined as those that had an average GIF, for both replicates of at least 2, which corresponds to 50% growth inhibition.

### Transcriptome and Deletome Data Analysis

An overview of the data analysis stream for both the transcriptome and the deletome data is shown in Figure S2. Briefly, GeneSpring v.7 (Agilent Technologies) and Rosetta Resolver v.3.2 (Rosetta Biosoftware, Seattle, WA) were used to identify genes that showed significant changes in gene expression with any metal treatment. The intensity ratio values from dye-swap hybridizations (individual metal concentration exposures and biological replicates separately) were combined using Rosetta Resolver error-weighted averaging and then assessed for significance of differential expression by computing p-values for the combined intensity ratio value for each gene [76],[77]. GeneSpring was used for global normalization of the raw microarray data using per feature and per chip and intensity dependent (LOWESS) normalization. Kruskal-Wallis test (p<0.05 with Bonferroni correction for multiple testing) of the genes under all experimental conditions (87,304 gene-treatment combinations; 6,236 genes×7 metals×2 concentrations) identified 4,296 significantly changed gene-treatments. A total of 1,341 genes were identified in the combined datasets (from the GeneSpring and the Rosetta Resolver analyses) that have at least a 2-fold change in the level of expression in at least 1 out of the 14 different metal treatment conditions.

Principal component analysis of the three biological replicates for each sample from all metal treatment conditions was performed using Partek Genomics Suite (Partek Incorporated, St. Louis, MO) and the intensity ratio data from the 1,341 genes. The first three principal components captured over 75% of the variability in the data.

Hierarchical and K-means clustering of the transcriptome and deletome were performed using Cluster 3.0 and visualized with Java TreeView 1.0.7 [78],[79]. K-means clustering on the average fold-change in gene expression values was performed with K = 6 for the genes, K = 3 for the samples Euclidean distance as the similarity metric. Gene Ontology (GO) analysis was performed using GO Term Finder (*Saccharomyces* Genome Database, Stanford, CA). GO categories in the transcriptome and deletome were also compared. Significant GO categories (Z-score≥2) for both profiles were obtained using GenMAPP 2.1 (Gladstone Institutes, San Francisco, CA). Z-scores for each GO term from this analysis were combined into a data matrix and subjected to hierarchical cluster analysis.

Cytoscape and the jActiveModule plug-in were used to identify neighborhoods in the regulatory networks associated with differentially expressed genes [25],[26]. In this analysis, the fold change and p-values of 6,236 genes under all treatment conditions from Rosetta Resolver were uploaded into Cytoscape. The p-values were processed as previously described [14],[25]. The yeast interaction network used in this analysis contained 5,604 protein modules with 22,574 protein-protein or protein-DNA interactions [80],[81].

## Supporting Information

Figure S1 Growth curves and viability assay of yeast exposed to metals. For growth measurements (left column): yeast were exposed to silver, arsenic cadmium, chromium copper, mercury and zinc at the indicated concentrations. Optical densities of the cultures were measured every 30 min. For viability assays (right column): yeast were exposed to the metals at the indicated concentrations for 1, 2 or 3 hr. The yeast were then collected, washed to remove the metal, and then plated on to fresh plates. The number of colony forming units (CFU) was determined following incubation at 30°C for 48 hr.
(2.20 MB TIF)

Figure S2 Flow chart describing data flow for transcriptome and deletome analyses.
(3.38 MB TIF)

Table S1 Fold-change, level of significance (p-values) and human orthologs for differentially expressed genes. The values of the clusters indicated in this table correspond to the information presented in Figure 2. Fold change and p-values were from three pairs of biological replicates and dye swap and were calculated using the Rosetta Resolver error model and error-weighted averaging method. Ensenbl gene identifiers and gene names known human orthologs of the differentially expressed yeast genes are presented.
(0.82 MB XLS)

Table S2 Gene Ontology terms and levels of significance for the genes presented in Figure 2. ^a^Corresponds to the clusters presented in Figure 2. ^b^Biological processes were identified using the Gene Ontology Term Finder tool in *Saccharomyces* Genome Database. ^c^Probability/significance values were calculated using the Gene Ontology Term Finder tool. ^d^Differentially expressed genes that are contained in the indicated Gene Ontology category.
(0.05 MB XLS)

Table S3 GIF's were calculated as described under Materials and Methods. ^a^Cluster values correspond to the clusters presented in Figure 3.
(0.13 MB XLS)

Table S4 Gene Ontology for the deletome data presented in Figure 3. ^a^Clusters correspond to the cluster designations presented in Figure 3. ^b^Biological processes and ^c^probabilities were calculated using the Gene Ontology Term Finder tool. ^d^Differentially expressed genes that are contained in the indicated Gene Ontology category.
(0.03 MB XLS)

Table S5 Genes and their corresponding Gene Ontologies, located in the overlapping regions of the Venn diagrams presented in Figure 4.
(0.02 MB XLS)

Table S6 Z-scores for Gene Ontology terms presented in Figure 5. Scores were calculated using GenMAPP.
(2.59 MB XLS)

## References

[1] Larocque ACL, Rasmussen PE (1998). An overview of trace metals in the environment, from mobilization to remediation.. Environ Geology.

[2] Cherrie JW, Semple S, Christopher Y, Saleem A, Hughson GW (2006). How important is inadvertent ingestion of hazardous substances at work?. Ann Occup Hyg.

[3] Goyer RA, Klaassen CD (2001). Toxic Effects of Metals.

[4] Valko M, Morris H, Cronin MT (2005). Metals, toxicity and oxidative stress.. Curr Med Chem.

[5] Stohs SJ, Bagchi D (1995). Oxidative mechanisms in the toxicity of metal ions.. Free Radic Biol Med.

[6] Ercal N, Gurer-Orhan H, Aykin-Burns N (2001). Toxic metals and oxidative stress part I: mechanisms involved in metal-induced oxidative damage.. Curr Top Med Chem.

[7] Harris GK, Shi X (2003). Signaling by carcinogenic metals and metal-induced reactive oxygen species.. Mutat Res.

[8] Tamás MJ, Labarre J, Toledano MB, Wysocki R (2006). Mechanisms of toxic metal tolerance in yeast..

[9] Chen F, Shi X (2002). Intracellular signal transduction of cells in response to carcinogenic metals.. Crit Rev Oncol Hematol.

[10] Zhao H, Eide D (1996). The yeast ZRT1 gene encodes the zinc transporter protein of a high-affinity uptake system induced by zinc limitation.. Proc Natl Acad Sci U S A.

[11] Liu XF, Culotta VC (1999). Post-translation control of Nramp metal transport in yeast. Role of metal ions and the BSD2 gene.. J Biol Chem.

[12] Vatamaniuk OK, Mari S, Lu YP, Rea PA (2000). Mechanism of heavy metal ion activation of phytochelatin (PC) synthase: blocked thiols are sufficient for PC synthase-catalyzed transpeptidation of glutathione and related thiol peptides.. J Biol Chem.

[13] Giaever G, Chu AM, Ni L, Connelly C, Riles L (2002). Functional profiling of the Saccharomyces cerevisiae genome.. Nature.

[14] Haugen AC, Kelley R, Collins JB, Tucker CJ, Deng C (2004). Integrating phenotypic and expression profiles to map arsenic-response networks.. Genome Biol.

[15] Gasch AP, Werner-Washburne M (2002). The genomics of yeast responses to environmental stress and starvation.. Funct Integr Genomics.

[16] Chattopadhyay MK, Tabor CW, Tabor H (2006). Polyamine deficiency leads to accumulation of reactive oxygen species in a spe2Delta mutant of Saccharomyces cerevisiae.. Yeast.

[17] Blaiseau PL, Lesuisse E, Camadro JM (2001). Aft2p, a novel iron-regulated transcription activator that modulates, with Aft1p, intracellular iron use and resistance to oxidative stress in yeast.. J Biol Chem.

[18] Casas-Finet JR, Hu S, Hamer D, Karpel RL (1992). Characterization of the copper- and silver-thiolate clusters in N-terminal fragments of the yeast ACE1 transcription factor capable of binding to its specific DNA recognition sequence.. Biochemistry.

[19] Liu Z, Boles E, Rosen BP (2004). Arsenic trioxide uptake by hexose permeases in Saccharomyces cerevisiae.. J Biol Chem.

[20] Venema J, Tollervey D (1999). Ribosome synthesis in Saccharomyces cerevisiae.. Annu Rev Genet.

[21] Kim M, Krogan NJ, Vasiljeva L, Rando OJ, Nedea E (2004). The yeast Rat1 exonuclease promotes transcription termination by RNA polymerase II.. Nature.

[22] Fauchon M, Lagniel G, Aude JC, Lombardia L, Soularue P (2002). Sulfur sparing in the yeast proteome in response to sulfur demand.. Mol Cell.

[23] Momose Y, Iwahashi H (2001). Bioassay of cadmium using a DNA microarray: genome-wide expression patterns of Saccharomyces cerevisiae response to cadmium.. Environ Toxicol Chem.

[24] Vido K, Spector D, Lagniel G, Lopez S, Toledano MB (2001). A proteome analysis of the cadmium response in Saccharomyces cerevisiae.. J Biol Chem.

[25] Ideker T, Ozier O, Schwikowski B, Siegel AF (2002). Discovering regulatory and signalling circuits in molecular interaction networks.. Bioinformatics.

[26] Shannon P, Markiel A, Ozier O, Baliga NS, Wang JT (2003). Cytoscape: a software environment for integrated models of biomolecular interaction networks.. Genome Res.

[27] Bernstein KA, Gallagher JE, Mitchell BM, Granneman S, Baserga SJ (2004). The small-subunit processome is a ribosome assembly intermediate.. Eukaryot Cell.

[28] Bernstein KA, Granneman S, Lee AV, Manickam S, Baserga SJ (2006). Comprehensive mutational analysis of yeast DEXD/H box RNA helicases involved in large ribosomal subunit biogenesis.. Mol Cell Biol.

[29] Martinez-Pastor MT, Marchler G, Schuller C, Marchler-Bauer A, Ruis H (1996). The Saccharomyces cerevisiae zinc finger proteins Msn2p and Msn4p are required for transcriptional induction through the stress response element (STRE).. Embo J.

[30] Heinisch JJ, Lorberg A, Schmitz HP, Jacoby JJ (1999). The protein kinase C-mediated MAP kinase pathway involved in the maintenance of cellular integrity in Saccharomyces cerevisiae.. Mol Microbiol.

[31] Beyer RP, Fry RC, Lasarev MR, McConnachie LA, Meira LB (2007). Multicenter study of acetaminophen hepatotoxicity reveals the importance of biological endpoints in genomic analyses.. Toxicol Sci.

[32] Bammler T, Beyer RP, Bhattacharya S, Boorman GA, Boyles A (2005). Standardizing global gene expression analysis between laboratories and across platforms.. Nat Methods.

[33] Kawata K, Yokoo H, Shimazaki R, Okabe S (2007). Classification of heavy-metal toxicity by human DNA microarray analysis.. Environ Sci Technol.

[34] Andrew AS, Warren AJ, Barchowsky A, Temple KA, Klei L (2003). Genomic and proteomic profiling of responses to toxic metals in human lung cells.. Environ Health Perspect.

[35] Hu Y, Wang G, Chen GY, Fu X, Yao SQ (2003). Proteome analysis of Saccharomyces cerevisiae under metal stress by two-dimensional differential gel electrophoresis.. Electrophoresis.

[36] Furst P, Hamer D (1989). Cooperative activation of a eukaryotic transcription factor: interaction between Cu(I) and yeast ACE1 protein.. Proc Natl Acad Sci U S A.

[37] Carri MT, Galiazzo F, Ciriolo MR, Rotilio G (1991). Evidence for co-regulation of Cu,Zn superoxide dismutase and metallothionein gene expression in yeast through transcriptional control by copper via the ACE 1 factor.. FEBS Lett.

[38] Causton HC, Ren B, Koh SS, Harbison CT, Kanin E (2001). Remodeling of yeast genome expression in response to environmental changes.. Mol Biol Cell.

[39] Yih LH, Peck K, Lee TC (2002). Changes in gene expression profiles of human fibroblasts in response to sodium arsenite treatment.. Carcinogenesis.

[40] Vujcic M, Shroff M, Singh KK (2007). Genetic determinants of mitochondrial response to arsenic in yeast Saccharomyces cerevisiae.. Cancer Res.

[41] Gasch AP, Spellman PT, Kao CM, Carmel-Harel O, Eisen MB (2000). Genomic expression programs in the response of yeast cells to environmental changes.. Mol Biol Cell.

[42] Warner JR (1999). The economics of ribosome biosynthesis in yeast.. Trends Biochem Sci.

[43] Moss T (2004). At the crossroads of growth control; making ribosomal RNA.. Curr Opin Genet Dev.

[44] Miller MJ, Xuong NH, Geiduschek EP (1982). Quantitative analysis of the heat shock response of Saccharomyces cerevisiae.. J Bacteriol.

[45] Neuman-Silberberg FS, Bhattacharya S, Broach JR (1995). Nutrient availability and the RAS/cyclic AMP pathway both induce expression of ribosomal protein genes in Saccharomyces cerevisiae but by different mechanisms.. Mol Cell Biol.

[46] Broach JR, Deschenes RJ (1990). The function of ras genes in Saccharomyces cerevisiae.. Adv Cancer Res.

[47] Markwardt DD, Garrett JM, Eberhardy S, Heideman W (1995). Activation of the Ras/cyclic AMP pathway in the yeast Saccharomyces cerevisiae does not prevent G1 arrest in response to nitrogen starvation.. J Bacteriol.

[48] Moskvina E, Schuller C, Maurer CT, Mager WH, Ruis H (1998). A search in the genome of Saccharomyces cerevisiae for genes regulated via stress response elements.. Yeast.

[49] Schmitt AP, McEntee K (1996). Msn2p, a zinc finger DNA-binding protein, is the transcriptional activator of the multistress response in Saccharomyces cerevisiae.. Proc Natl Acad Sci U S A.

[50] Gorner W, Durchschlag E, Martinez-Pastor MT, Estruch F, Ammerer G (1998). Nuclear localization of the C2H2 zinc finger protein Msn2p is regulated by stress and protein kinase A activity.. Genes Dev.

[51] Sutherland DJ, Tsang BK, Merali Z, Singhal RL (1974). Testicular and prostatic cyclic amp metabolism following chronic cadmium treatment and subsequent withdrawal.. Environ Physiol Biochem.

[52] Gunnarsson D, Nordberg G, Lundgren P, Selstam G (2003). Cadmium-induced decrement of the LH receptor expression and cAMP levels in the testis of rats.. Toxicology.

[53] Portela P, Moreno S (2006). Glucose-dependent activation of protein kinase A activity in Saccharomyces cerevisiae and phosphorylation of its TPK1 catalytic subunit.. Cell Signal.

[54] Santangelo GM (2006). Glucose signaling in Saccharomyces cerevisiae.. Microbiol Mol Biol Rev.

[55] Zurita-Martinez SA, Cardenas ME (2005). Tor and cyclic AMP-protein kinase A: two parallel pathways regulating expression of genes required for cell growth.. Eukaryot Cell.

[56] Charron MJ, Dubin RA, Michels CA (1986). Structural and functional analysis of the MAL1 locus of Saccharomyces cerevisiae.. Mol Cell Biol.

[57] Michels CA, Read E, Nat K, Charron MJ (1992). The telomere-associated MAL3 locus of Saccharomyces is a tandem array of repeated genes.. Yeast.

[58] Bandhakavi S, McCann RO, Hanna DE, Glover CV (2003). Genetic interactions among ZDS1,2, CDC37, and protein kinase CK2 in Saccharomyces cerevisiae.. FEBS Lett.

[59] Canton DA, Litchfield DW (2006). The shape of things to come: an emerging role for protein kinase CK2 in the regulation of cell morphology and the cytoskeleton.. Cell Signal.

[60] Rudra D, Mallick J, Zhao Y, Warner JR (2007). Potential interface between ribosomal protein production and pre-rRNA processing.. Mol Cell Biol.

[61] Tang F, Liu G, He Z, Ma WY, Bode AM (2006). Arsenite inhibits p53 phosphorylation, DNA binding activity, and p53 target gene p21 expression in mouse epidermal JB6 cells.. Mol Carcinog.

[62] Martin DE, Soulard A, Hall MN (2004). TOR regulates ribosomal protein gene expression via PKA and the Forkhead transcription factor FHL1.. Cell.

[63] Chuang SM, Wang IC, Yang JL (2000). Roles of JNK, p38 and ERK mitogen-activated protein kinases in the growth inhibition and apoptosis induced by cadmium.. Carcinogenesis.

[64] Kim SH, Bark H, Choi CH (2005). Mercury induces multidrug resistance-associated protein gene through p38 mitogen-activated protein kinase.. Toxicol Lett.

[65] Samet JM, Graves LM, Quay J, Dailey LA, Devlin RB (1998). Activation of MAPKs in human bronchial epithelial cells exposed to metals.. Am J Physiol.

[66] Schaeffer HJ, Weber MJ (1999). Mitogen-activated protein kinases: specific messages from ubiquitous messengers.. Mol Cell Biol.

[67] Brewster JL, de Valoir T, Dwyer ND, Winter E, Gustin MC (1993). An osmosensing signal transduction pathway in yeast.. Science.

[68] Posas F, Wurgler-Murphy SM, Maeda T, Witten EA, Thai TC (1996). Yeast HOG1 MAP kinase cascade is regulated by a multistep phosphorelay mechanism in the SLN1-YPD1-SSK1 “two-component” osmosensor.. Cell.

[69] Raitt DC, Posas F, Saito H (2000). Yeast Cdc42 GTPase and Ste20 PAK-like kinase regulate Sho1-dependent activation of the Hog1 MAPK pathway.. Embo J.

[70] Begley TJ, Rosenbach AS, Ideker T, Samson LD (2002). Damage recovery pathways in Saccharomyces cerevisiae revealed by genomic phenotyping and interactome mapping.. Mol Cancer Res.

[71] Birrell GW, Brown JA, Wu HI, Giaever G, Chu AM (2002). Transcriptional response of Saccharomyces cerevisiae to DNA-damaging agents does not identify the genes that protect against these agents.. Proc Natl Acad Sci U S A.

[72] Parsons AB, Brost RL, Ding H, Li Z, Zhang C (2004). Integration of chemical-genetic and genetic interaction data links bioactive compounds to cellular target pathways.. Nat Biotechnol.

[73] Memarian N, Jessulat M, Alirezaie J, Mir-Rashed N, Xu J (2007). Colony size measurement of the yeast gene deletion strains for functional genomics.. BMC Bioinformatics.

[74] Brachmann CB, Davies A, Cost GJ, Caputo E, Li J (1998). Designer deletion strains derived from Saccharomyces cerevisiae S288C: a useful set of strains and plasmids for PCR-mediated gene disruption and other applications.. Yeast.

[75] Jang YK, Jin YH, Kim MJ, Seong RH, Hong SH (1995). A simple and efficient method for the isolation of total RNA from the fission yeast Schizosaccharomyces pombe.. Biochem Mol Biol Int.

[76] Hughes TR, Marton MJ, Jones AR, Roberts CJ, Stoughton R (2000). Functional discovery via a compendium of expression profiles.. Cell.

[77] Stoughton R, Dai H (2002). Statistical combining of cell expression profiles. USA..

[78] de Hoon MJ, Imoto S, Nolan J, Miyano S (2004). Open source clustering software.. Bioinformatics.

[79] Saldanha AJ (2004). Java Treeview–extensible visualization of microarray data.. Bioinformatics.

[80] Lee TI, Rinaldi NJ, Robert F, Odom DT, Bar-Joseph Z (2002). Transcriptional regulatory networks in Saccharomyces cerevisiae.. Science.

[81] Xenarios I, Salwinski L, Duan XJ, Higney P, Kim SM (2002). DIP, the Database of Interacting Proteins: a research tool for studying cellular networks of protein interactions.. Nucleic Acids Res.

