# Supplementary figures and images for: Global Transcriptome and Deletome Profiles of Yeast Exposed to Transition Metals
Source: PLoS Genet. 2008 Apr 25;4(4):e1000053. doi: 10.1371/journal.pgen.1000053 (PMC2278374; doi:10.1371/journal.pgen.1000053)

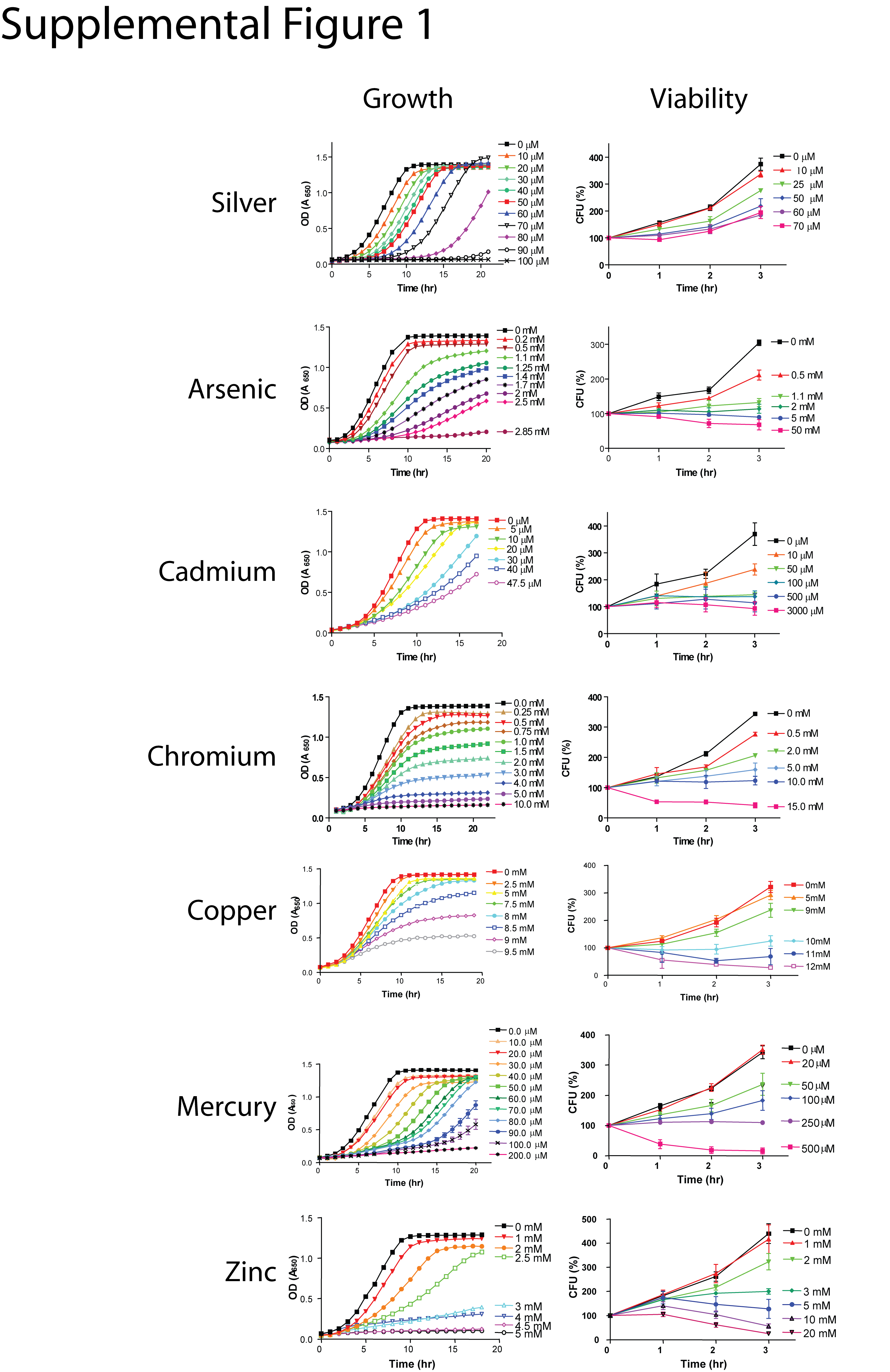

Supplement: Figure S1 — Growth curves and viability assay of yeast exposed to metals. For growth measurements (left column): yeast were exposed to silver, arsenic cadmium, chromium copper, mercury and zinc at the indicated concentrations. Optical densities of the cultures were measured every 30 min. For viability assays (right column): yeast were exposed to the metals at the indicated concentrations for 1, 2 or 3 hr. The yeast were then collected, washed to remove the metal, and then plated on to fresh plates. The number of colony forming units (CFU) was determined following incubation at 30°C for 48 hr. (2.20 MB TIF) [file pgen.1000053.s001.tif]

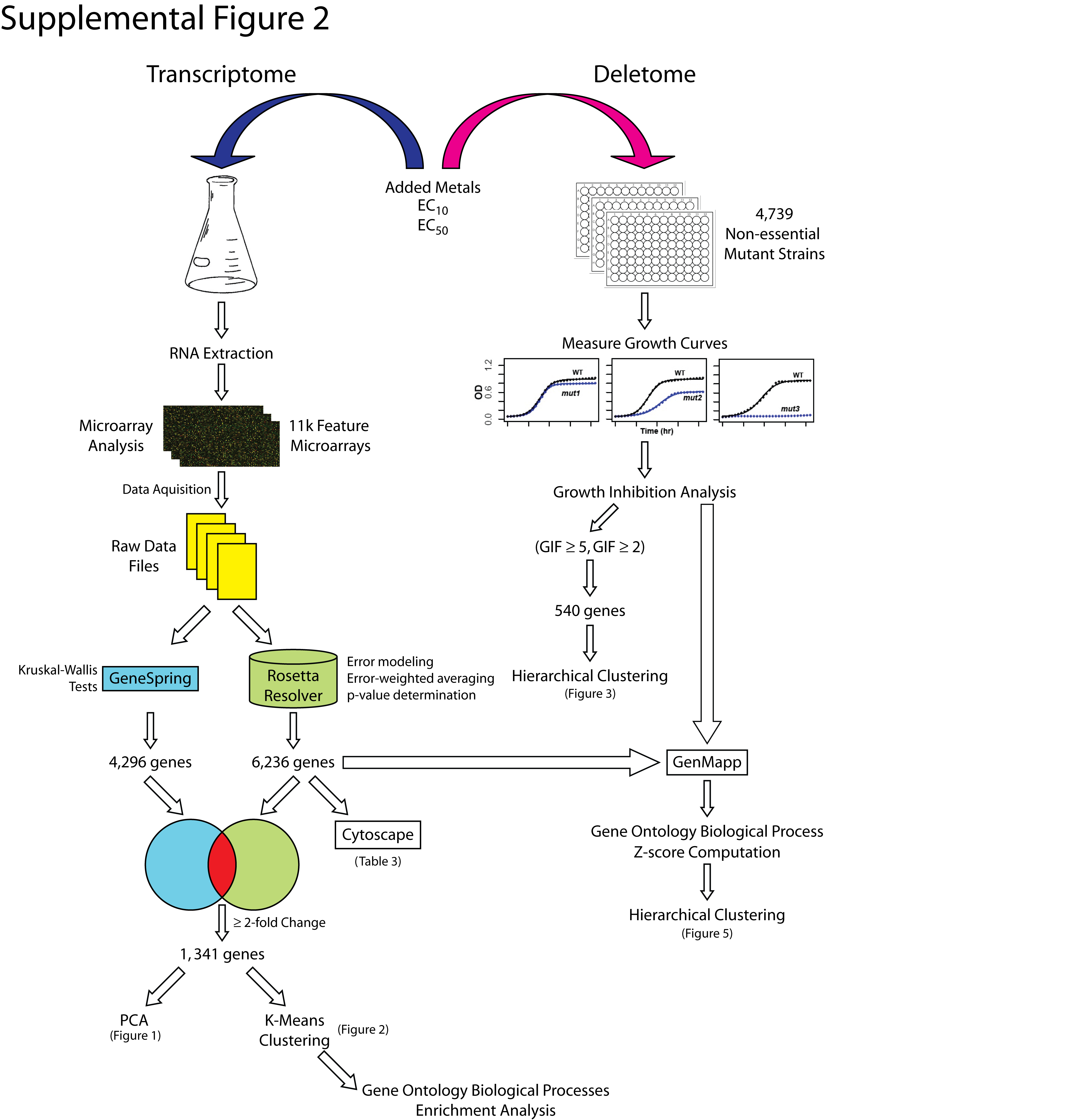

Supplement: Figure S2 — Flow chart describing data flow for transcriptome and deletome analyses. (3.38 MB TIF) [file pgen.1000053.s002.tif]
